# The Case for Day Nurseries

**Published:** 1915-03-27

**Authors:** 


### MATERNITY AND MOTHERING.

### The Case for Day Nurseries.

BY AN ACTING PRESIDENT OF A DAY NURSERY AND MOTHERS' SCHOOL.

We cannot fail to realise at the present moment that the children are now the country's most valuable asset. It behoves us, therefore, to try to find some unfailing, effectual, and permanent means of arresting the terrible wastage of infant life that is going on in our large cities.

It must have struck many already that when our soldiers return from the Front with shattered limbs we shall be called upon to take care of the children, and thus leave our women at liberty to earn their daily bread, free from anxiety about their offspring. We can, and we must, help the mothers; and it can be done in the following practical form.

### Why Nurseries are Needed.

We want nurseries in every part of the kingdom, so that mothers can go to work feeling that their babies are in safe hands, in wholesome surroundings, with fresh air and good food and sanitation, and receiving the necessary medical supervision. It does not require much foresight to grasp the fact that, more than ever, women are about to become the breadwinners; and, therefore, to cater for the children becomes a necessity.

Babies, it may be said, are born strong and healthy; yet, for the lack of simple knowledge, they often become rickety and anæmic—if, indeed, they survive the neglect to which, through ignorance, they are subjected.

Thousands of infants die, or, if they live, they help to fill our "Homes for Crippled Children"—useless, unhappy members of society who, had they been properly nurtured at the beginning of their existence, would have become healthy girls and boys. These "Homes" are filled only with the children of the poor; for where do we find a "Home for Cripples" for the children of the better classes?

The first step is to teach our girls (at as early an age as may seem advisable) the simple rules of mothercraft. They must learn to wash, dress, and undress and bathe a baby, and be taught something of its minor ailments and what to do in case of accidents. Several experiments of this description are being tried, and "Schools for Mothers" are being opened for the purpose of instructing the elder pupils of our elementary schools.

### The Leeds Experiment.

At Leeds the experiment is already proving of much value, and at the "Day Nursery" classes are held every morning of the week, Saturdays excepted.

There the girls, after a course of lessons which include practical demonstrations, are allowed to undress, bathe, dress, and then feed a baby. Notes are taken, and an examination follows in due course.

Far be it from any of us to encourage women with children to go out to work, for is it not a mother's first work to stay at home to suckle her infant, to look after her offspring, and to see to her husband's comfort and her house? But what of the widow, the deserted woman, the unmarried wife, and the woman whose husband is in feeble health, away, or in gaol? These women are compelled to work, and they have children to provide for. In our anxiety to help the old people are we to forget the rising generation? We have old-age pensions, almshouses, homes for the aged; why no nurseries for our babies?

What, so far, are we doing, and what is the best that happens to the children when the mother is reluctantly compelled to go out and earn her daily bread? The child is either left in the care of an old grannie, far too feeble and much too old and ignorant to take proper charge of it, or it is put into the hands of a neighbour, who probably drinks, and during the time she frequents the nearest public-house her charge falls into the fire and gets badly burnt; in many such cases, too, a child is given insufficient or improper food, the woman's sole object being to make as much as possible out of her bargain.

### The French Creche.

Other countries are far ahead of us in this respect. France takes the lead. For in Paris, just forty years ago, crèches were first opened for the use and benefit of the children of the working women of the city. There are now over five hundred in the country, half of which are municipalised, and it is in France that the ideal crèche is found.

In many of the large factories where women are employed the owner generously gives up a room, or rooms, for this purpose, provides a suitable staff and all the other requirements necessary, and the mothers have the privilege given them to come at regular hours and suckle their infants. In Germany, in Holland, in Denmark, and in Sweden such nurseries are common, while in Toronto several are also doing good service.

It is a fact that our Local Government Board and our Board of Education are recognising the necessity for nurseries and similar institutions for the children of the working poor, by giving assistance and grants to those associations that are doing useful work on sensible lines, but much more help is needed, more universal co-operation. As yet the work has been left entirely to voluntary workers, supported solely by voluntary contributions. Voluntary efforts, valuable in a thousand different directions, need every encouragement, but we do want greater interest and larger funds if we are to succeed, and there must be no stint of either.

The National Society of Day Nurseries, founded in 1906 by Sir John Gorst—well known for his love of children—has done wonders to improve the conditions of day nurseries. Besides inspecting them annually it allows grants to those that are proving satisfactory, and at its central organisation substantial help is given to those who are desirous of starting new ones. The Society again has raised the general standard by refusing to recognise any nursery as efficient unless it is considered good enough to be affiliated to the National Society.

Small nurseries, though decidedly more costly, are certainly preferable, not only because the children can be given greater care and more individual attention, but because children should not be herded together in large numbers. In these nurseries the tiniest child can be trained to acquire decent habits and to develop good manners. Indeed it is now a recognised fact that children who have been trained at a good nursery are easily picked out by the teachers of the elementary schools.

A small charge, varying from 2d. to 6d. per day, according to circumstances, is paid by the mothers, and for this sum a child is fed and kept clean and warm and comfortable during the long day when the mother is at her work.

In many of the more recently opened crèches those women who suckle their infants can go to them during the mill dinner-hour for that beneficial purpose, and are provided with a hot, substantial meal for the modest sum of 4d., a meal which must prove of great service to their health and comfort. Again, clothing clubs are open where the women can buy sensible, reasonable garments for themselves and their babies at a very modest cost.

A good matron is ever ready (when the mothers come to the nursery) to offer friendly advice to them on the management of their own health and on that of their infants. Arrangements are often made for a matron to have enough spare time to visit the women in their own homes, so that if a child fails to put in a regular appearance she can discover the reason. If the cause be illness, advice and suitable food or milk are given for a short time, or arrangements can be made for the child to be admitted to a convalescent home or hospital.

If there is reason to suppose the mothers are not well, or in any difficulty, sympathetic workers will often visit them, and such visits by trained visitors are appreciated. In many instances a medical man gives his services at a crèche, and in paying a weekly visit he can inspect all the children and advise the matron about their health.

It is becoming quite the custom for young girls to seek training in nurseries, starting as probationers, where, by going through a course of mothercraft, they learn to fit themselves for suitable posts, as such experience stands them in good stead later on. "Nursery nurses" can also undergo a short or long course of training by arrangement.

